# Spectral Identification of Lighting Type and Character

**DOI:** 10.3390/s100403961

**Published:** 2010-04-20

**Authors:** Christopher D. Elvidge, David M. Keith, Benjamin T. Tuttle, Kimberly E. Baugh

**Affiliations:** 1 Earth Observation Group, Solar and Terrestrial Division, NOAA National Geophysical Data Center, 325 Broadway, Boulder, CO 80305 USA; 2 Marshall Design Inc., Boulder, CO, USA; E-Mail: david.keith@mindspring.com; 3 Cooperative Institute for Research in Environmental Science, University of Colorado, Boulder, Colorado 80303, USA; E-Mails: ben.tuttle@noaa.gov (B.T.T.); kim.baugh@noaa.gov (K.E.B.); 4 Department of Geography, University of Denver, Denver, CO, USA

**Keywords:** lighting types, lighting efficiency, photopic band, nighttime lights, Nightsat, LED

## Abstract

We investigated the optimal spectral bands for the identification of lighting types and the estimation of four major indices used to measure the efficiency or character of lighting. To accomplish these objectives we collected high-resolution emission spectra (350 to 2,500 nm) for forty-three different lamps, encompassing nine of the major types of lamps used worldwide. The narrow band emission spectra were used to simulate radiances in eight spectral bands including the human eye photoreceptor bands (photopic, scotopic, and “meltopic”) plus five spectral bands in the visible and near-infrared modeled on bands flown on the Landsat Thematic Mapper (TM). The high-resolution continuous spectra are superior to the broad band combinations for the identification of lighting type and are the standard for calculation of Luminous Efficacy of Radiation (LER), Correlated Color Temperature (CCT) and Color Rendering Index (CRI). Given the high cost that would be associated with building and flying a hyperspectral sensor with detection limits low enough to observe nighttime lights we conclude that it would be more feasible to fly an instrument with a limited number of broad spectral bands in the visible to near infrared. The best set of broad spectral bands among those tested is blue, green, red and NIR bands modeled on the band set flown on the Landsat Thematic Mapper. This set provides low errors on the identification of lighting types and reasonable estimates of LER and CCT when compared to the other broad band set tested. None of the broad band sets tested could make reasonable estimates of Luminous Efficacy (LE) or CRI. The photopic band proved useful for the estimation of LER. However, the three photoreceptor bands performed poorly in the identification of lighting types when compared to the bands modeled on the Landsat Thematic Mapper. Our conclusion is that it is feasible to identify lighting type and make reasonable estimates of LER and CCT using four or more spectral bands with minimal spectral overlap spanning the 0.4 to 1.0 um region.

## 1. Introduction

“How many economists does it take to change a light bulb? None — market forces change light bulbs.”

**Figure 1.**
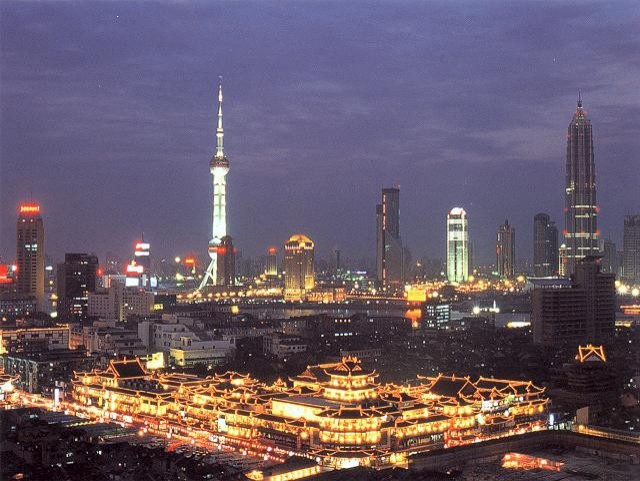
Shanghai, China at night.

Humans are unique in being the only organism on the planet that produces lighting using external sources. Over time there have been a series of transitions in lighting types, as technological advances made it possible for lighting to be provided at lower cost to larger numbers of people. In ancient times lighting was obtained by burning dry vegetation. Gradually open fires were replaced by candles and lamps fueled by animal and mineral oils. Natural gas was widely used as a lighting source in the 1880s. In 1880 Thomas Edison patented an electric light suitable for commercialization. Regarding the prospects for electric lighting Edison said “We will make electricity so cheap that only the rich will burn candles”. Indeed electric lighting predominates today and our cities are bathed in light well into the night (Figure 1).

Edison’s development was the commercial incandescent lamp, which operates by heating a tungsten filament to emit light. Today incandescent light bulbs account for 79% of all light bulb sales, but only 8% of the usable light [1]. The large number of sales can be attributed to the low cost, simplicity of installation and rapid lifecycle (typically 800–1,000 hours) of the incandescent bulb. The low percentage of usable light provided by incandescent lights arises from the fact more than 80% of the energy emitted by incandescent bulbs is in the infrared, outside the range of human vision. Over time electric lighting types diversified, a process driven by demand for large area lighting, cost efficiency, visual attractions (e.g., neon lighting) and the preference for color renderings that are similar to daylight. Incandescent lights still dominate both interior and exterior residential lighting. However, gas discharge lamps (e.g., fluorescent, metal halide, and high pressure sodium) predominate in most other settings. Gas discharge lamps generate light by passing electric arcs through chambers containing gases which glow at specific wavelengths (emission lines) as electrons shift orbital shells. Gas discharge lamps are more efficient than incandescent light bulbs since many of their primary emissions are in the visible portion of the spectrum. Additionally, phosphors are used to shift emissions to desired wavelengths. Fluorescent lights dominate office interior lighting but are also used extensively for outdoor lighting in countries such as India. Mercury vapor lamps, metal halide lamps and high pressure sodium lamps dominate for large area lighting, including interiors of building with high ceilings, exteriors of non-residential buildings, street and road lighting. Liquid fuel lamps (e.g., kerosene) are the primary lighting source for approximately 1.6 billion people who lack access to electricity [1,2].

Phospors are mineral compounds that absorb radiation of certain wavelengths and then re-radiate at longer wavelengths for a short period. The application of phosphors in fluorescent and induction lamps allows for the conversion of ultraviolet radiation from mercury vapor arcs into visible radiation. Some mercury vapor and metal halide lamps are also coated with phosphors. Some LED sources also use phosphors. A typical approach to producing “white” light from LEDs is to combine a blue LED (peak ∼460 nm) with a phosphor coating to produce a broad spectrum in the green-yellow-red range. The manufacturer is able adjust the balance between these two components to produce a range of correlated-color temperatures (CCTs) for “white” LEDs.

The potential for future cost savings and carbon emission reductions achievable through replacing inefficient lamps and fixtures is enormous. Worldwide lighting consumes an estimated 19% of all electric power, more than all the nuclear and hydro-power plants combined [1]. The total cost of lighting worldwide is estimated to be $460 billion per year (2005), with 75% of this cost attributed to electricity [1]. Total annual carbon emissions associated with lighting have been estimated at 1889 MtCO_2_ [1]. During the next several decades one can anticipate a substantial transformation in lighting types worldwide–with the widespread installation of long-lived energy efficient lights, installation of shields to restrain the escape of light into the sky, and installation of controls to dim or shut off lights when no one is present. These transformations will be driven primarily by societal moves towards cost efficiency and reductions in carbon emissions to the atmosphere. However, health and environmental rationales [3] for changes in lighting may also play a role in lighting decisions. A recent manifestation in the long history of energy-based transformation in lighting has already started, with more than a billion incandescent light bulbs having been replaced by CFLs over the past decade. Over the long term, LEDs have the potential to become the predominant lighting type because of their continuing development towards extremely low energy consumption, long life, and color variability [4]. In addition, there is a synergism between implementation of renewable power generation systems and the installation of low power LED lighting that can be powered by locally generated wind and solar power [2].

The detection of lights from space has been possible with data from the Defense Meteorological Satellite Program (DMSP) since the early 1970s [5]. While global in extent, the DMSP collects low light imaging data in a single spectral band straddling the visible and near infrared (NIR), making it impossible to discriminate lighting types or the spectral quality of lights. In addition, DMSP nighttime lights are acquired at coarse spatial resolution (2.7 km^2^) with six bit quantitization and no on-board calibration. The DMSP sensor is typically operated at high gain setting for the detection of moonlit clouds. As a result, the signal often saturates on bright urban cores. Despite these flaws, a diverse set of global applications have been developed for DMSP nighttime lights ranging from estimation of light pollution levels [6], constructed surface densities [7], population distribution [8], poverty rates [9], electrification rates [10], and estimates of resource consumption rates [11].

Based on the shortcomings of DMSP nighttime lights Elvidge and others developed a Nightsat mission concept [12,13], which outlines the spatial resolution, spectral band options, overpass time and repeat cycle considerations for future satellite sensors capable of collecting global nighttime lights for use in a variety of social, energy and environmental applications. Key recommendations for Nightsat include a spatial resolution in the range of 50 meters, multiple spectral bands for the discrimination of lighting types, and a repeatable in-flight radiance calibration procedure. The Nightsat papers recommended inclusion of the photopic [14] and scotopic [15] human visual sensitivity bands based on the fact that these are widely used by the lighting engineering community. In particular, lighting systems are designed to provide specific brightness levels in the photopic band for different types of human activities. However, the recommendation to include the photopic and scotopic spectral bands on Nightsat had not been based on evidence that these spectral bands were useful in discriminating lighting type or character.

A skeptic could say it will be impossible to identify lighting types from space because most pixels will be a murky mixture of light from multiple lighting types, that variable atmospheric scatter and absorption will distort the signal, and that light reflected off of ground objects will be altered by the absorption characteristics of these materials. These factors are also issues for daytime remote sensing of the Earth’s surface, a field which has been active and successful since the 1972 launch of the first Landsat satellite. Over the years, the atmospheric correction of satellite remote sensing data has become quite advanced, through the use of radiative transfer models such as MODTRAN [16]. The spectral purity of a Nightsat pixel will be dependent on the number and relative proportion of discrete lighting types found in or adjacent to the pixel footprint. Here some compromise must be sought since it is not practical to observe individual lights from space. At the other spatial resolution extreme, it is clear that coarse resolution imagery, such as DMSP, would often have substantial mixing of lighting types. The key is to set the spatial resolution small enough that it is close to the spatial resolution with which lighting types vary, yielding a preponderance of pixels where a single lighting type dominates the signal.

**Figure 2.**
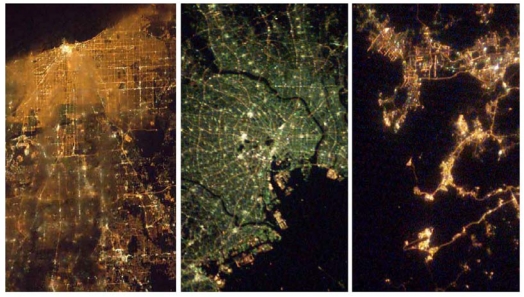
Color camera images of Chicago, Tokyo and Hong Kong acquired at sixty meter resolution from the International Space Station. Note the presence of optically thin clouds in the Chicago image, resulting in a blur of the street pattern.

For proof that it is possible to collect spectral information on lights from space we offer the “cities at night” digital color camera images collected by astronauts from the International Space Station. Figure 2 shows ISS images of Chicago, Tokyo and Hong Kong collected by Donald R. Pettit [17]. The images clearly show different colors of lights and spatial detail never seen in the DMSP nighttime lights. The Chicago image shows golden orange (high pressure sodium lamps?), brown, green and white colored lights. The Tokyo image is dominated by green lights (metal halide lamps?), with some major highways and port areas showing orange lights, and isolate patches of white lighting. Hong Kong has many orange lights, with minor areas of green, white and magenta lights. In each case the spatial resolution is sufficient to resolve the internal structure of the city, linear patterns of streets and roads, plus individual clusters of development. Note how the thin clouds present in the Chicago image blurs the pattern of the streets and roads, but does not affect the color of the detected lighting in an obvious fashion. Perhaps as important as revealing where lights are present–the images also reveal where detectable lighting is absent. The purity of the golden orange lights of Chicago and the emerald green lights of Tokyo suggests that at 60 meter resolution there is substantial purity of the spectra of light observed from space and that variations in the reflectance of buildings, trees, grass, streets, roads, sidewalks, cars and trucks appear to have little spectral impact on the light escaping to space from these cities.

The dramatic cities at night imagery from the ISS indicate that it is possible to observe the spectral character of outdoor lighting from space, even with a standard color camera. This leads to questions that drive our investigation: What type of information could be obtained from space with a sensor equipped with spectral bands optimized for nighttime lights? Would it be possible to discriminate different lighting types? Would it be possible to rate the efficiency of installed lighting and estimate the electric power consumption? Would it be possible to rate the character or spectral quality of the lighting? Would it be possible to track conversions to more cost efficient lighting types?

In this study we assemble a library of high spectral resolution laboratory emission spectra of primary lighting types and analyze these spectra vis-a-vis the questions listed above. The concept is that what is possible with laboratory spectra may also be possible with data collected with a satellite sensor, with appropriate detection limits, sufficient spatial resolution, and atmospheric correction. Conversely, if we find that laboratory spectra perform very poorly for estimating a particular variable–there is very little prospect that this variable could be derived at a usable accuracy from a satellite sensor. In this approach we follow in the footsteps of other scientists who had their eyes on eventual satellite remote sensing, but started out with field and laboratory spectra [18–21] to establish spectral libraries and determine the primary spectral phenomena that could exploited via remote sensing. We use the spectral library to evaluate the utility of the human visual sensitivity bands *versus* alternative spectral band sets for their ability to discriminate lighting types, to assess lighting efficiency with two indices (luminous efficacy (LE) and luminous efficacy of radiation (LER)) and two widely used indices for lighting character (correlated color temperature (CCT) and color rendering index (CRI)). Governments are increasingly setting standards for the LE, CCT and CRI of installed lighting [1]. The objective of our study is to provide an evidence based recommendation for a future Nightsat sensor capable of producing spatially explicit survey maps of lighting type, lighting quantity and lighting character worldwide. We believe that such maps would be useful in accelerating the conversion to more efficient lighting types and tracking the progress of lighting type conversions in a cost effective manner.

## 2. Methods

### 2.1. Collection of Emission Spectra

Emission spectra were acquired using an ASD, Inc. FieldSpec 3 spectroradiometer equipped with an 8 degree field of view foreoptic. The instrument had been radiometrically calibrated (NIST traceable) and spectra were acquired in radiance (Watts/m^2^/sr/nm) mode over the 350 to 2,500 nm range at one nm resolution. The instrument has three spectrometers: 350 nm to 1,000 nm (VNIR), 1,001 nm to 1,830 nm (SWIR 1), and 1,831 nm to 2,500 nm (SWIR 2). ASD reports the spectral resolution is 3 nm (full width half maximum) in the VNIR spectrometer and 10 nm in the two SWIR spectrometers. The instrument sampling interval is 1.4 nm from 350 to 1,050 and 2 nm from 1,050 to 2,500. Post processing yields spectra with consistent 1 nm increments from 350 to 2,500 nm. Thus, there is a radiance reported at each nanometer over the entire measured range. The start and warm up of each lamp was visually observed to ensure that the lamp was operating at a stable state prior to measurement. Each lamp was warmed up for at least one hour or more prior to measurement and the spectra were acquired from one lamp at a time in an otherwise-dark room.

The measured light sources included the following classes and are described in Table 1: 1) liquid fuel lamps, 2) pressurized fuel lamps, 3) incandescent (including quartz halogen), 4) mercury vapor, 5) fluorescent, 6) metal halide, 7) high pressure sodium, 8) low pressure sodium, and 9) light emitting diodes (LED).

### 2.2. Processing to Spectral Bands

**Figure 3.**
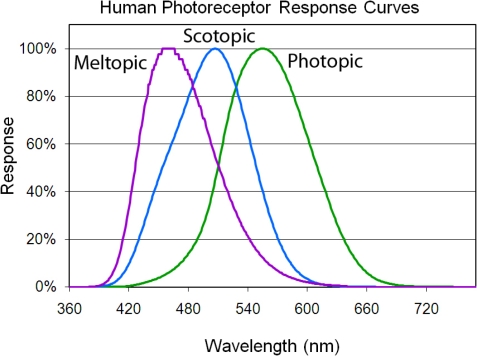
Spectral response functions of the three human photoreceptor bands.

The high spectral resolution emission spectra were processed to simulate the radiances in eight broad spectral bands. This processing involved multiplying the radiance at each wavelength by the spectral band’s response (0.0 to 1.0) at that wavelength and then summing the results. The simulated radiances are thus weighted for the shape of the spectral band’s response function. The simulated broad bands include three spectral functions defined based on the wavelength-dependent sensitivities of the human eye (Figure 3) and five spectral bands defined based on remote sensing considerations. The photopic and scotopic spectral bands are widely used in the lighting science and engineering communities and are recognized by the International Commission on Illumination (CIE) [14,15]. The photopic band is a representation of the combined wavelength-dependent sensitivities of the cone photoreceptors in the human eye. These are the three types of photoreceptors that together provide color vision under suitably bright conditions. The scotopic band is a representation of the sensitivity of the rod photoreceptors, which provide black and white vision under dim lighting conditions, also referred to as night-vision. The photopic and scotopic bands have maximum sensitivity at 555 (green) and 507 nm (blue-green) respectively. In the late 1990’s a third photoreceptor was recognized in the human eye [22,23] which has sensitivity in the blue spectral range. This is a non-imaging photoreceptor which provides stimulation to the daytime portion of the 24-hour circadian cycle or biological clock. The “meltopic” band is defined based on the wavelength-dependent suppression of melatonin production [24,25]. Research has found that exposure to bright white or blue light at night is associated with insomnia and increased risk for a wide range of diseases [26–30].

**Figure 4.**
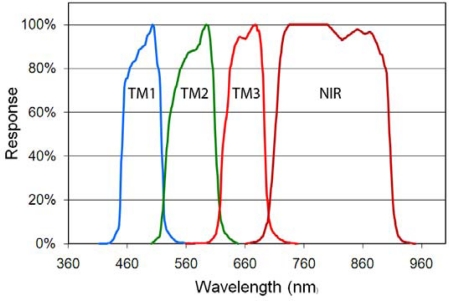
Set of four spectral bands based on the Landsat Thematic Mapper (TM). Note that the short wavelength edge of the near infrared TM band (NIR) was shifted to 700 nm. An additional orange spectral band was formed by merging the green (TM 2) and red (TM 3) bands.

As can be seen in Figure 3 the human visual and non-visual sensitivity spectral bands do not extend into the near infrared and have substantial overlap in their wavelength sensitivities. A set of spectral bands with minimal spectral overlap and coverage in the near-infrared may be superior for the discrimination of lighting type and character. Rather than devise a new set of spectral bands we decided to work from the visible and near infrared spectral band set of the Landsat Thematic Mapper (TM) based on the spectral positions of the bands [31], the minimal overlap between the bands, and their compatibility with cost effective sensor design and construction. We did not simulate the short wavelength infrared (SWIR) spectral bands of Thematic Mapper since detectors for imaging sensors in these wavelength regions are quite expensive. We extended the short wavelength edge of the TM4 near infrared (NIR) band to eliminate the gap that exists between the third and fourth TM bands. The result is a set of four spectral bands: blue, green, red and NIR (Figure 4). The spectra were also processed through an orange spectral band formed by merging TM bands two and three. The orange band was introduced to explore the performance of a three band system covering the same spectral range as the first four TM bands. The rationale being that a three band satellite sensor could be built at a lower cost than a four band system and three band data would be easier to view and process than four band data.

For the analysis of lighting type and character using the broad spectral bands we defined the eight band combinations listed in Table 2. The first three sets are combinations of the photoreceptor bands, starting with the photopic band by itself and adding one spectral band with each step. Sets four and five combine the photopic band with the non-green TM bands. Set six is a three band combination with TM bands 1, 2 and 3. Set seven is a four band combination with TM bands 1, 2, 3 plus the broad NIR band. Set eight is a three band combination with TM band 1, an orange band formed by merging TM bands 2 and 3, plus the NIR band. These band combinations could be viewed as candidates spectral band set for a Nightsat sensor having no more than four discrete spectral bands. As such, they can be rated based on their performance in discriminating lighting types and the estimation of luminous efficacy (LE), luminous efficacy of radiation (LER), correlated color temperature (CCT) and color rendering index (CRI).

## 3. Results

### 3.1. Spectral Characteristics of the Lamps

**Figure 5.**
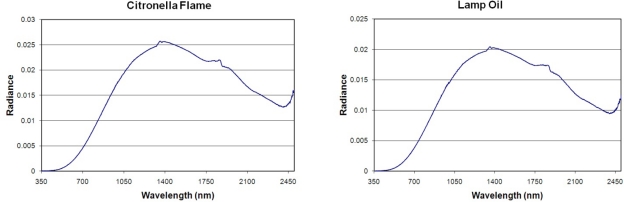

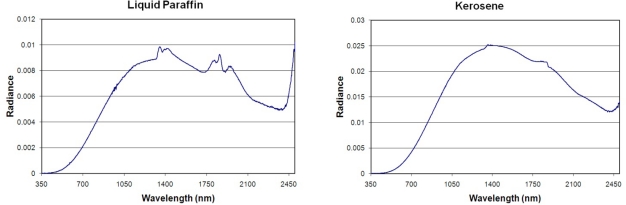
Emission spectra of lamps burning liquid fuels largely exhibit blackbody curves.

**Liquid Fuel Lamps:** Emission spectra were collected from four liquid fuel lamps (citronella oil, lamp oil, liquid paraffin, and kerosene). The spectra (Figure 5) largely follow blackbody curves with peaks near 1,350 nm and an upward swing in emission from 2,400 to 2,500 nm. The liquid paraffin spectrum has a distinctive set of infrared emission on top of the blackbody curve, with features from 1,300 to 1,450 nm and from 1,700 to 2,000 nm. The higher upswing in emission from 2,400 to 2,500 in the liquid paraffin spectrum suggests that this is the short wavelength edge of a stronger emission feature in the 3,000 nm range.

**Figure 6.**
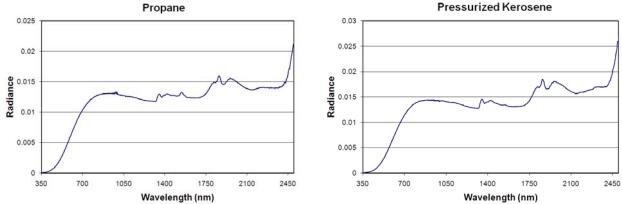
Emission spectra of lanterns burning pressurized fuel.

**Pressurized Fuel Lamps:** Spectra were acquired from pressurized fuel lamps equipped with mantles burning kerosene and propane. The mantles contain rare earth oxides which absorb infrared radiation to glow white in the visible. When compared to the liquid fuel flames, the pressurized lamp spectra have a flattened appearance, with the short wavelength leading edge of the blackbody curve shifted deeper into the visible (Figure 6). The pressurized gas flames have a similar set of emission features observed in the liquid paraffin spectrum from 1,300 to 1,450 nm and 1,700 to 2,000 nm, plus the upswing from 2,400 to 2,500 nm also seen in all the liquid flame spectra.

**Figure 7.**
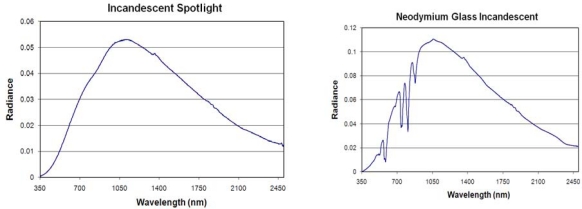
Emission spectra of incandescent lamps.

**Incandescent Lamps:** Spectra were acquired from four incandescent lamps and two quartz halogen lamps. These lamps have tungsten filaments inside a glass bulb that contains either a vacuum or an inert gas to prevent oxidation of the hot filament. The emission spectra of incandescent lamps have blackbody shapes (Figure 7). The peak radiance occurs at shorter wavelengths (900 to 1,050 nm) than the liquid fuel spectra and have none of the infrared emission features seen in the pressurized kerosene, propane, and liquid paraffin spectra. It is possible to alter the emission pattern with a pigment coating on the bulb or by altering the composition of the glass enclosure. A common example of this is the introduction of the rare earth element neodymium to the glass, which produces a series of absorptions centered at 572, 737, 806 and 877 nm (Figure 7).

**Figure 8.**
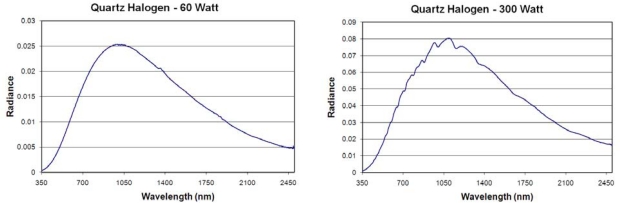
Emission spectra of quartz halogen lamps.

One of the shortcomings of the standard tungsten filament incandescent lamp is that over time the interior of the bulb tends to become coated with tungsten which has ablated off the filament. The gradual loss of tungsten is a leading cause for failure of the standard incandescent lamp. In the quartz halogen lamp the tungsten filament is sealed in a compact transparent quartz inner envelope filled with an inert gas and a small amount of a halogen such as iodine or bromine. Quartz halogen lamps make use of a chemical reaction called the halogen cycle to redeposit the tungsten back onto the filament. This allows the lamp to operate at a higher temperature and yields a longer service life. The emission spectra of the measured quartz halogen lamps (Figure 8) are very similar to the standard incandescent lamps, with emission peaks in the 970 to 980 range and a luminous efficiency in the range of 15 to 20%, comparable to the LE of the incandescent lamps. One of the quartz halogen lamp spectrum exhibits a series of shallow absorptions in the 700 to 1,400 nm range. These are likely due to a trace element present in the glass used in the bulb, similar to the neodymium bulb. There were no emission or absorption features noted that could distinguish standard incandescent from quartz halogen lamps.

**Figure 9.**
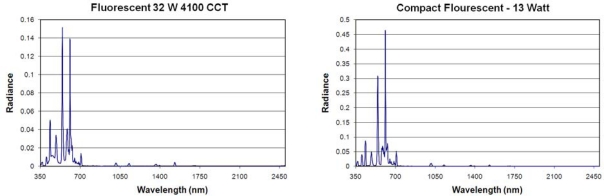
Emission spectra of a standard fluorescent tube and an compact fluorescent light (CFL).

**Figure 10.**
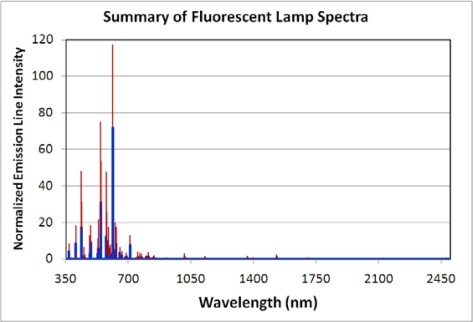
Spectral variability of fluorescent lamps. Each of the nine spectra were normalized to 1.0 and then an average (blue) and standard deviation (red) were calculated.

**Fluorescent Lamps:** Spectra were acquired from nine fluorescent lamps, including two compact fluorescent lamps. The fluorescent lamp is a low-pressure gas discharge lamp that generates light predominately by phosphors excited by UV emissions. The glass tube is filled with a mixture of low pressure mercury vapor and inert gases such as argon, xenon, neon, or krypton. Electrons in the vapor are excited by an electric arc to produce a combination of visible and ultraviolet (UV) emissions. The primary mercury emissions in the ultraviolet are non-visual but can damage eyes. To redistribute the UV emitted radiation into the visible, the inner surface of the glass tube is coated with a fluorescent coating made of metallic and rare-earth phosphor salts. The fluorescent lamp spectra consist of a set of sharp emission lines (Figure 9). To summarize the emission features and variability found in fluorescent lamp spectra, we normalized each spectrum by dividing each spectrum by its maximum radiant emission and then calculating the mean and standard deviation from the nine normalized spectra (Figure 10). This revealed that fluorescent lamps have two primary emission lines at 544 and 611 nm, with the line at 611 nm usually the stronger of the two. In Figure 10, if the 611 nm line was the strongest for every one of the nine normalized spectra, it would show up with a normalized peak height of 100 - however, since this line is the strongest for some but not all of these spectra, it ends up with a normalized emission line intensity of 72 and has a standard deviation of 45 on that scale. Other strong emission lines occur at 546, 436, and 545 nm. There is substantial variability in the intensity of emissions at 611, 544, 574, 546, 436, 545, 578, 437 and 530 nm. The infrared emissions are quite low.

**Figure 11.**
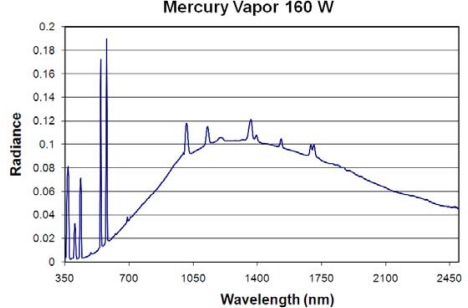
Emission spectrum of a mercury vapor lamp.

**Mercury Vapor Lamp:** This is a high intensity discharge (HID) lamp which uses an electric arc to excite mercury to produce light. In contrast to fluorescent lamps, the arc discharge is confined to a small fused quartz tube mounted within a larger borosilicate glass bulb that is in this case coated with a phosphor that absorbs the UV emissions and fluoresces, producing more light in the visible range. We measured a single mercury vapor lamp (Figure 11). This is a self-ballasted variety with a spotlight shape, with a reflective coating on the base of the outer bulb (spotlight style). This lamp produces a substantial quantity of heat and its spectrum bears some resemblance to an incandescent lamp, with a blackbody peak near 1,260 nm. The primary emission lines are at 546 and 578 nm. Secondary emission lines are at 366, 403, 435, 1,012, 1,125, 1,362, 1,525, 1,688 and 1,692 nm.

**Figure 12.**
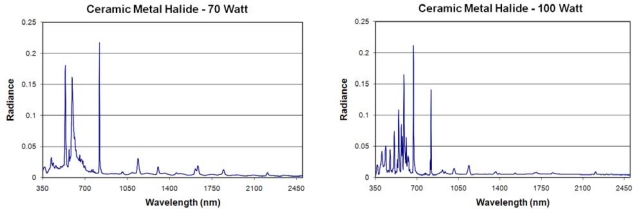

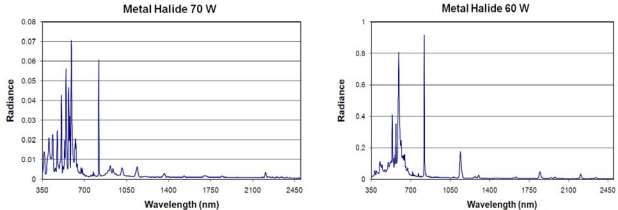
Emission spectra of four metal halide lamps.

**Figure 13.**
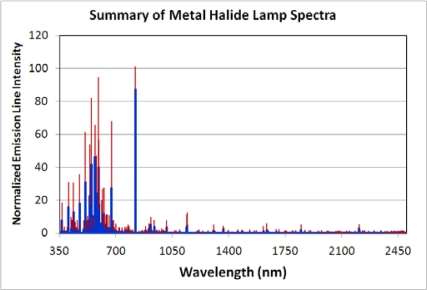
Spectral variability of metal halide lamps. Each of the four spectra were normalized to 1.0 and then an average (blue) and standard deviation (red) were calculated.

**Metal Halide Lamps:** These high intensity discharge (HID) lamps are similar to mercury-vapor but improved to produce different spectra by mixing a variety of metal halides into the mercury vapor. The composition of halides present determines the location and intensity of the emission lines, making it possible for metal halide lamps to range in appearance from “cool white” to “warm white”. We measured the spectra of four metal halide lamps (Figure 12) and analyzed the variability (Figure 13). Each of the metal halide lamps had a strong emission at 819 nm, location of a known set of narrow sodium emission lines. In two of the four spectra the 819 nm line was the strongest emission. One lamp had its strongest emission line at 671 nm—an emission line also present in the other metal halide spectra. Other strong emission lines were found at 569, 547, 591, 509, 671, 578, 536, 474, 593, 537, 405 and 590 nm. The bands having the most variability (in descending order) are 591, 671, 547, 593, 537, 590, 594, and 509 nm. In addition to the major emission line at 819 nm, infrared emissions are present at 915, 937, 1,013, 1,139, 1,365, 1,634, 1,846 and 2,207 nm.

**Figure 14.**
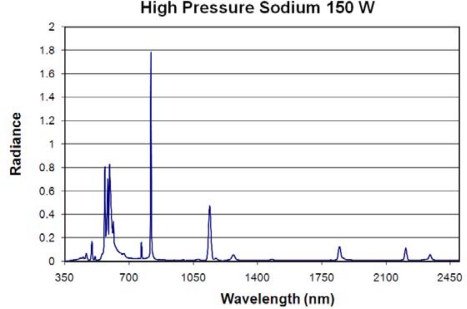
Emission spectrum of a high pressure sodium lamp.

**Figure 15.**
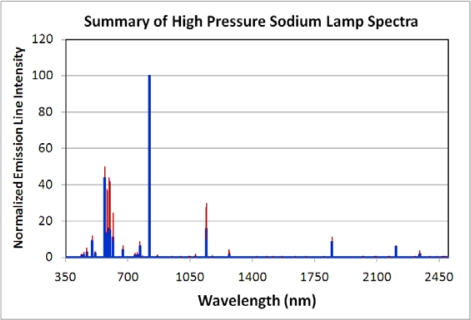
Spectral variability of metal halide lamps. Each of the three spectra were normalized to 1.0 and then an average (blue) and standard deviation (red) were calculated.

**High Pressure Sodium Lamps:** These are HID lamps containing a sodium-mercury amalgam and trace quantities of inert gas, such as xenon, to assist in the startup. An electric arc passing through the chamber excites the electrons on the sodium and mercury atoms, causing them to glow. These lamps produce a characteristic golden-orange light. Spectra were measured for three high pressure sodium (HPS) lamps. The strongest emission line is from the set of sodium emissions at 819 nm (Figure 14). This emission line is also present in the metal halide lamp spectra. Other strong emission lines occur at 569, 594, 1,140, 595, and 598 nm. There is a dense cluster of strong emission lines from 569 to 616 nm. In addition to the 819 and 1,140 nm lines, there are infrared emission lines at 767, 1,269, 1,846, 2,207, and 2,339 nm. The mean and standard deviation analysis found that the most variable emission line is at 594, followed by the emission lines at 595, 598, 582, 585, 584, 1,140 and 615 nm (Figure 15). Overall, the HPS spectra have less variability than the fluorescent and metal halide lamps.

**Figure 16.**
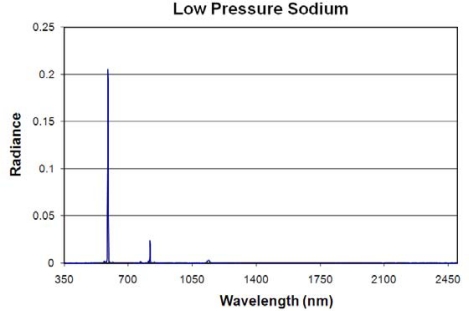
Emission spectrum of a low pressure sodium lamp.

**Low Pressure Sodium Lamps:** Low pressure sodium (LPS) lamps produce light by exciting sodium vapor and feature an outer vacuum envelope of glass coated with an infrared reflecting layer which reduces the emission of infrared light. The result is a single strong sodium emission line at 589 nm and a much smaller secondary sodium emission line at 819 nm (Figure 16). The resulting light is a distinctive orange color.

**Figure 17.**
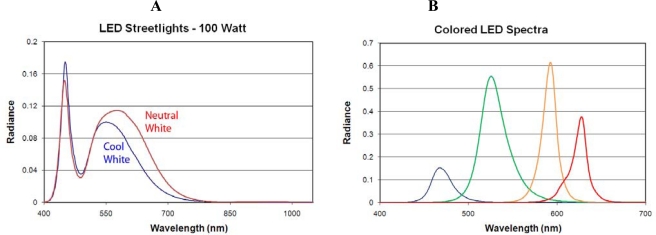
Spectra of LED lamps. A) Comparison of two white LED streetlights. B) Spectra from four colored LEDs.

**Light Emitting Diodes (LED):** Spectra were acquired from thirteen LED lamps. These are solid-state light sources that generate light by electroluminescence, moving electrons from a high energy state to a lower energy state on a semi-conductor substrate. We measured a variety of LEDs with appearance ranging from white to blue, green, orange and red. Figure 17a shows two spectra from white LED streetlights featuring the primary emission at 450–460 nm and the phosphor induced secondary emission in the green and into the red. The spectrum from the neutral white lamp has stronger emission in the red when compared to the cool white LED. Figure 17b shows the color variability that is possible with LEDs. The LED spectra are typified by relatively narrow emission bands and virtually no NIR emission. All of the white LED spectra we measured have two overlapping emission bands, the result of coat a blue LED with a phosphor layer. The measured LED lamps had virtually no emission beyond 800 nm.

### 3.2. Discrimination of Lighting Types

**Figure 18.**
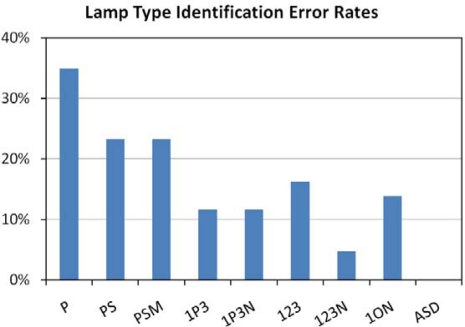
Error rates for the identification of nine types of lights.

The ability to correctly identify lighting type was evaluated for the eight sets of spectral bands (Table 2) and the full resolution ASD spectra. The analysis was done using the discriminant analysis available in the JMP statistical package [32]. The 43 spectra were divided into nine types: liquid fuel, pressurized fuel, incandescent (including quartz halogen), mercury vapor, fluorescent, low pressure sodium, high pressure sodium, metal halide, and LED. There were zero errors in the identification of lighting types using the full resolution ASD spectra (Figure 18). The next best result came from the four bands Thematic Mapper (with the modified near-infrared band) band set, with an error rate of 4.7%. The band combinations with the photopic plus TM bands had error rates of 11.6%. The first three TM bands and the three band set with TM band 1, orange, and NIR yielded 13.9 and 16.3% error rates respectively. The combinations with only the human visual and non-visual sensitivity bands had substantially higher error rates for the identification of lighting type.

### 3.3. Estimation of Luminous Efficacy

**Figure 19.**
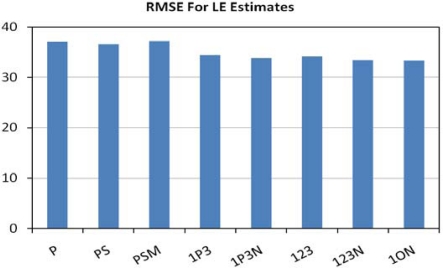
Error rates for estimation of luminous efficacy.

**Figure 20.**
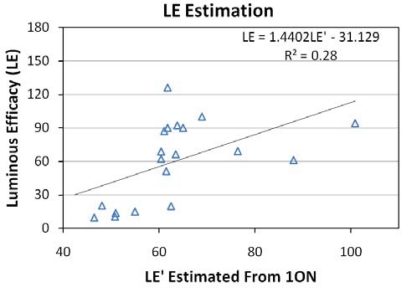
LE estimated from the 1ON band combination *versus* the LE calculated from manufacturer reported lumen output and consumption watts.

Luminous efficacy (LE) is a primary figure of merit for a lamp that rates the quantity of usable light produced per watt of electric power consumption. Usable light is defined as the total output in the photopic spectral band in lumens (weighted radiance times 683). Since the ASD instrument only measured a portion of the radiant output of each lamp, we relied on manufacturer data on output lumens and power consumption to calculate luminous efficacy. It was only possible to find this data for about half of the measured lamps. The data sources used are listed in a spreadsheet available at http://www.ngdc.noaa.gov/dmsp/nightsat.html. Linear regression was used to analyze the relationship between the radiances and LE values. The band combinations were then rated based on the Root Mean Square Error (RMSE) for the estimation of LE. The lowest root mean square error (RMSE) came from the three band combination of the photopic band with TM bands 1 and 3 (Figure 19), with an RMSE of 33.4. As can be seen in Figure 20, the ability to estimate LE with this band combination is poor, yielding an R^2^ of 0.28. Our assessment, based on the data in hand, is that LE cannot be accurately estimated with spectral techniques.

### 3.4. Luminous Efficacy of Radiation (LER):

**Figure 21.**
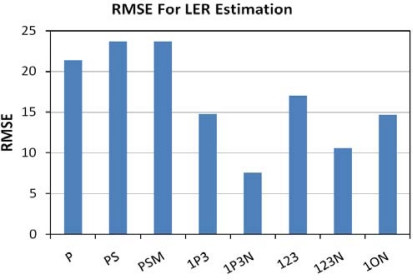
RMSE for LER estimates.

**Figure 22.**
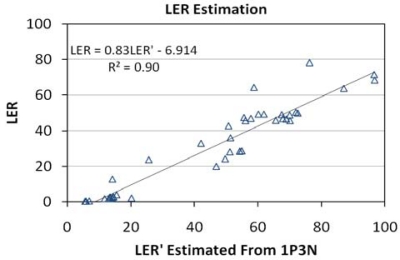
LER *versus* estimated LER from TM1, photopic, TM3 and NIR bands.

LER is the percentage of emitted radiance that is useful for human vision, as defined by the photopic band. This is a measure of the efficiency of lighting, but is not as useful as luminous efficacy (LE), which can be used to estimate electric power consumption. The high spectral resolution emission data were used to calculate the true LER of each lamp by dividing the simulated radiance in the photopic band by the total measured radiance from 350 to 2,500 nm. LER was estimated for each of the Table 2 band combinations by dividing the photopic band radiance (or TM band 2 in the combinations that lacked the photopic band) by the sum of the radiances in each of the bands. The ability of the band combinations to estimate LER was analyzed with linear regression. Figure 21 shows the RMSE values for the tested band combinations. The lowest RMSE comes from the band combination using the photopic band plus the TM bands 1, 3 and the NIR band (IP3N). Figure 22 shows the 1P3N estimated LER *versus* the actual values and the relationship is quite good, yielding and R^2^ of 0.90. The 1P3N band combination spans the 450 to 900 nm range and has the advantage of including the precise numerator (photopic band radiance) for the calculation of the LER. The errors in the estimation are likely due to the fact that these bands provide no information on the radiance in the 350–450 nm and 900–2,500 nm ranges. It should be noted that the band combination 123N was only slightly.

### 3.5. Correlated Color Temperature (CCT):

This is a standard rating of the apparent color temperature of a lamp in degrees Kelvin. The simplest case is for the incandescent light, where the emission pattern is driven by their temperature (blackbody behavior). For these lamps the wavelength dependent emission can be predicted using Planck’s Law. The wavelength with the maximum radiance is dependent on the temperature of the emitting object and shift to shorter wavelengths as temperature increases (Weins’ Displacement Law). Thus as an object’s temperature increases it will begin to glow red and gradually shift to yellow and white as the temperature continues to rise. Incandescent lights typically have color temperatures in the 2,700 to 3,300 Kelvin range. For gas discharge and LED lamps the emissions are focused in the visible and do not follow a black-body curve. However, it is still useful to estimate their “color” by calculating the CCT, which is the color temperature as perceived by the human eye. These CCT values are used to guide the selection of lamps for specific environments.

**Figure 23.**
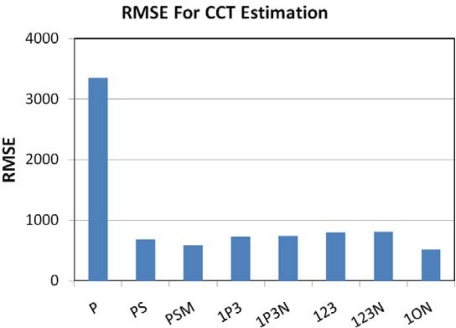
RMSE values for CCT estimation.

**Figure 24.**
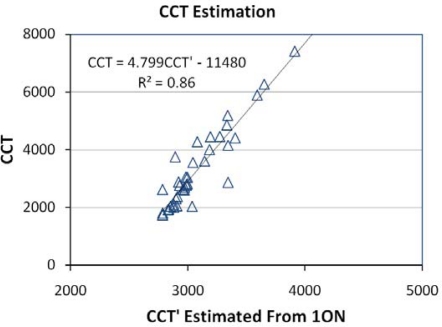
The 1ON estimates of CCT’ *versus* the CCT calculated from the ASD spectra. Linear regression yields an R^2^ of 0.86.

For the CCT analysis we used the color space described by CIE [33] and the algorithm outlined by Wyszecki and Stiles [34]. The high spectral resolution emission data were used to calculate the true CCT for each lamp. CCT was estimated for each of the Table 2 band combinations using the CIE algorithm. The ability of the band combinations to estimate CCT was evaluated based on the root mean square errors between the estimated and true CCT values. Figure 23 shows the RMSE values for the estimation of CCT from the Table 2 band combinations. All of the multiband combinations yielded similar RMSE values, but the lowest RMSE came from the TM 1, orange and NIR band combination (1ON). The 1ON estimates of CCT *versus* the CCT calculated from the ASD spectra are shown in Figure 24. Linear regression yielded an R^2^ of 0.86.

### 3.6. Color Rendering Index (CRI):

**Figure 25.**
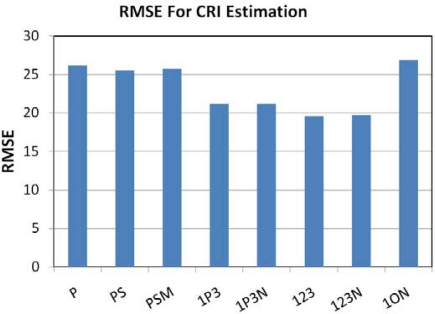
RMSE for CRI estimation. The lowest RMSE came from the TM band 1,2,3 combination.

**Figure 26.**
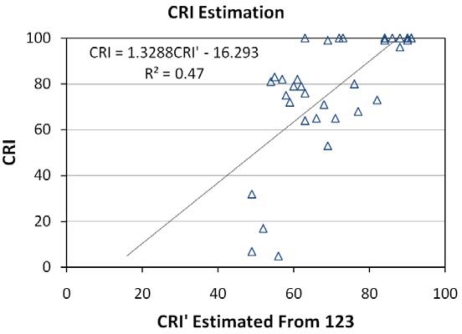
CRI *versus* estimated CRI’ using TM bands 1, 2 and 3.

This is a measure of the ability of a light source to reproduce the colors faithfully in comparison to a blackbody radiator with matching color temperature. Incandescent lights typically yield high CRI values, near 100. Low pressure sodium lamps are nearly monochromatic, yielding very poor color rendition. CRI values were calculated for each lamp with the CIE method [35] using the high spectral resolution emission data as the input. The ability of the band combinations to estimate CRI was analyzed using linear regression and evaluated based on the root mean square errors. Figure 25 shows the RMSE values for each of the band combinations. The lowest RMSE comes from the TM band 1,2,3 combination. Figure 26 shows the band 1,2,3 estimated CRI *versus* actual CRI values. Linear regression yielded and R^2^ of 0.47. The results indicate that high spectral resolution is required for accurate calculation of CRI.

## 4. Conclusion

While there is much to be gained through the conversion of lighting types and fixtures, to date there is no systematic mechanism to inventory lighting type, lighting efficiency or the quantity of lighting worldwide. Such information, in map form and periodically updated, could be used to accelerate the installation of more efficient lamps and fixtures. Astronaut photography of cities at night confirm that satellite remote sensing could be used to make detailed maps of outdoor lighting and light escaping from buildings through windows, doors and skylights. But this would require a specialized low light imaging sensor capable of distinguishing lighting types, lighting character and measuring the quantity of light escaping to space.

To investigate the optimal spectral bands to accomplish these objectives we collecting high spectral resolution emission spectra, from 350 to 2,500 nm, for forty-three different lamps, encompassing nine of the major types of lamps used worldwide. This spectral library is available at: http://www.ngdc.noaa.gov/dmsp/nightsat.html. We found substantial variation in the emission spectra of lighting types. Lamps that produce light through heat (incandescent, quartz halogens, and fuel lamps) emit primarily like blackbodies, with peak emission in the near infrared and emissions higher in the red than green and blue. Fluorescent, metal halide, high pressure and low pressure sodium lamps are gas discharge lamps, which emit different series of narrow emission lines. The identity of the gas discharge lamps can be discerned based on the wavelength positions of the emission lines. Fluorescent lamps have a pair of very strong mercury emissions at 544 and 611 nm. Outside of these two strong emissions lines there is substantial variability in the secondary emission lines from fluorescent lamps in the visible range and virtual no emission in the infrared out to 2,500 nm. Metal halide lamps have a highly variable set of emission lines in the visible, but always have strong emission at 819 nm and a set of tightly packed emissions lines centered at 569 nm. High and low pressure sodium vapor lamps have very little variability. Both have strong sodium emission lines at 819 nm. The low pressure sodium lamp has only one additional emission line of any consequence, at 589 nm. The high pressure sodium lamp has a series of emission lines from 569 to 616 nm and a set of minor emission lines in the blue region. The emission spectra of LED’s are highly variable—but characteristically have symmetrically shaped emission peaks (resembling Gaussian curves) and extremely low emission in the infrared out to 2,500 nm. The white LED’s we measured all had two such emission peaks, a primary emission peak in the blue and a second peak in the green to red. The second emission peak in the white LED spectrum is induced by a phosphor coating on the LED which absorbs a portion of the blue emission and reradiates at longer wavelengths.

For the identification of lighting types it is clear that a hyperspectral sensor would be ideal. However a hyperspectral sensor with the required detection limits capable of acquiring global coverage at moderate spatial resolution (20 to 50 meters) would be very costly. We found that the Landsat Thematic Mapper bands (with a slight modification to the NIR band) performed nearly as well in the identification of lighting types as the full resolution hyperspectral data acquired by the ASD spectroradiometer. With the bandset based on the TM there was spectral confusion between fluorescent lamps and white LED lamps. The human eye photoreceptor bands (photopic, scotopic and “meltopic”) did not perform well for the identification of lighting types. We attribute this to the extensive overlap in the bandpasses of these bands and the lack of response to NIR light.

For the general purpose of distinguishing lighting types and lighting character, there does not appear to be great value in having spectral bands beyond 1,000 nm. The LED’s we measured had zero emission beyond 800 nm. Gas discharge (fluorescent, metal halide, high pressure sodium) have only minor emissions beyond 830 nm. Incandescent, quartz halogen, mercury vapor, and fuel base lighting have substantial emission in the 1,000 to 2,500 nm range, but this blackbody emission pattern can be well established at wavelengths short of 1,000 nm. Sensors operating under 1,000 nm are able to use silicon based CCD detectors, which are considerable less expensive than the arrays of mercury-cadmium-telluride (HgCdTe) or indium-gallium-arsenide (InGaAs) detectors that operate in the 1,000 to 2,500 nm range. Thus our recommendation is that the Nightsat spectral band selections focus on the visible and near-infrared short of 1,000 nm.

We analyzed the variability found in the emission lines from fluorescent, metal halide and high pressure sodium lamps. The high pressure sodium lamps had very little variability. In contrast, the metal halides exhibited substantial variability in both the presence or absence of specific emission lines and their relative intensities. The variability of the fluorescent lamp emission lines was not as high as for metal halides. These findings suggest that additional examples of both fluorescent and metal halide lamps should be added to the spectral library. In addition, these results indicate that the setting used for the spectral identification of metal halide and fluorescent lamps should have more tolerance for variation than setting designed for the identification of high pressure sodium lamps.

**Figure 27.**
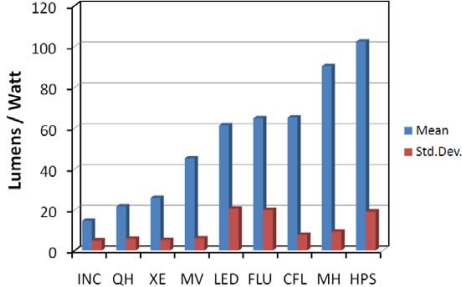
Mean and standard deviation for luminous efficacies calculated for a wide range of lamps derived from manufacturer data. INC = incandescant, QH = quartz halogen, XE = xenon arc, MV = mercury vapor, LED = light emitting diode, FLU = linear fluorescent, CFL = compact fluorescent, MH = metal halide, HPS = high pressure sodium.

None of the spectral band combinations performed well at the estimation of luminous efficacy (LE), which is a measure of the photopic output per watt of input electricity. LE integrates the inefficiencies that each style of lamp has in converting electricity into photopic light, such as the efficiency of the ballast for fluorescent and HID lamps, heating of lamps and fixtures, energy lost through the use of phosphor coatings, and emission of light outside the photopic band. Other than the emission of light outside the photopic band, these are variables that cannot be measured spectrally in the 350 to 2,500 nm range. However, LE can be reasonably estimated based on lighting type (Figure 27). This suggests that accurate identification of lighting types may be the key to eventual surveys of lighting efficiency from space.

For three of the spectral metrics for the quality of light emitted by lights (LER, CCT and CRI) it can be fairly said that hyperspectral data are the standard source for the calculation. For LER the best broad band combination included the photopic band plus bands in the blue, red and NIR (1P3N). CCT was reasonably estimated with the blue, orange and NIR bands (1ON). None of the band combinations performed well in the estimation of CRI.

We have identified a dilemma regarding the inclusion of the photopic band on a Nightsat sensor. Including the photopic band improves the accuracy of the LER estimates. In addition, inclusion of the photopic band could be useful in estimating human activity levels or economic activity levels since lighting installations are designed to provide specific fluxes of light in the photopic band. However, the photoreceptor bands are of little value in the identification of lighting types due to the extensive overlap in their spectral response curves. The four band set based on the TM proved to be better than the photoreceptor bands for the identification of lighting types and the TM bands are capable of making reasonable estimates of CCT, LER and MPR. The solution to the dilemma is to add more spectral bands, either including the photopic band along with a band set similar to the TM or by subdividing the visible range into a set of (five or six) bands that are useful for identifying lighting types and can be used to simulate the radiances in the photopic band. Splitting the TM bands would likely improve the ability to identify lighting types and could enable the simulation of the results from photopic band, enabling improved estimation of LER. For instance, if the visible range is split into five spectral bands, ranging from blue to red, the three bands in the middle could be used to estimate the radiance in the photopic band. We found that including at least one band in the NIR to be quite useful for distinguishing incandescent sources from other lighting types and in estimating LER. But there may be some advantage to splitting the NIR into two spectral bands, including the 819 nm sodium emission from high pressure sodium and metal halides in the short wavelength NIR band and having a second NIR band running from 850 to 950 nm. This could enhance the discrimination of high intensity discharge (HID) lamps from fluorescent and LED lamps.

While our study is specifically aimed at the eventual satellite remote sensing of lighting type and character, the results are relevant to the selection of instrumentation for ground based surveys. Given the modest cost of field portable spectroradiometers, there is very little reason to consider using broad band spectral devices for field surveys if the objective is to survey the installed base of lighting types and how this changes over time.

In conclusion, we find that it is possible to identify lighting types, the efficiency and quality of lighting using spectral data that could be acquired from space. With a low light imaging hyperspectral sensor it would be possible to identify lighting types proportions of lighting types through spectral mixture analysis, and estimate luminous efficacy of radiation (LER), correlated color temperature (CCT), and color rendering index (CRI). While a hyperspectral sensor would be the best option, this would be technically challenging and quite costly. We investigated what could be achieved with a limited number (1–4) of broad spectral bands and found with four spectral bands, modeled on the first four TM bands, it is possible to identify lighting types with few errors and to also make reasonable estimates of LER and CCT. It is likely that the performance achieved with of the broad band approach can be improved by subdividing the visible and near infrared bands and this should be a topic for future research.

## Figures and Tables

**Table 1.** Lighting Types Measured.

	**Type**	**Description**	**CCT**	**CRI**	**Lumens**	**Watts**	**LE**
1	Liquid Fuel	Citronella Oil	1941	100			
2	Liquid Fuel	Kerosene	1913	100			
3	Liquid Fuel	Lamp Oil	1935	99			
4	Liquid Fuel	Liquid Paraffin	2038	100			
5	Pressurized Fuel	Pressurized Kerosene	2308	96			
6	Pressurized Fuel	Propane	2380	100			
7	Incandescent	100 Watt Spot	2709	99			
8	Incandescent	100 Watt Neodymium	2604	73	960	100	9.6
9	Incandescent	200 Watt clear	2826	100	3980	200	19.9
10	Incandescent	52 Watt frosted	2632	100	710	52	14.0
11	Quartz Halogen	300 Watt double ended	2775	100	6100	300	20.3
12	Quartz Halogen	60 Watt standard base	2788	100	900	60	15.0
13	Fluorescent	13 Watt compact	2886	81	900	13	69.2
14	Fluorescent	9 Watt compact	2766	83	550	9	61.1
15	Fluorescent	4100 CCT (a)	4009	79	2950	32	92.2
16	Fluorescent	High CRI	4279	80			
17	Fluorescent	Low CRI	4415	5			
18	Fluorescent	5000 CCT	5193	76			
19	Fluorescent	4100 CCT (b)	4452	82			
20	Fluorescent	3500 CCT	3560	75			
21	Fluorescent	3000 CCT	3056	82			
22	High Pressure Sodium	150 W	2056	17	16000	170	94.1
23	High Pressure Sodium	1000 W	2108	32	126000	1000	126.0
24	High Pressure Sodium	70 W	2005	7	6300	70	90.0
25	Low Pressure Sodium	18 W SOX	1807		1570	18	87.2
26	Metal Halide	100 W	3610	65	9000	100	90.0
27	Metal Halide	70 W	3049	79	7000	70	100.0
28	Metal Halide	90 W (a)	4160	64	5600	90	62.2
29	Metal Halide	90 W (b)	2874	100	6200	90	68.9
30	Mercury Vapor	Self Ballasted	3758	53	3000	160	10.5
31	LED	Spotlight	5899	65	250	9	27.8
32	LED	Streetlight (A)	4863	71	5580	109	51.2
33	LED	Amber Standard Base	8357				
34	LED	Green Standard Base	7272				
35	LED	Red Standard Base	1756				
36	LED	Blue Tester	6592	83			
37	LED	Green Tester	3124	72			
38	LED	Orange Tester	1739				
39	LED	Red Tester	2624	68			
40	LED	White Tester	7419				
41	LED	Yellow Tester	2038	99			
42	LED	Streetlight (Cool White)	6273	100	3604	54.3	66.4
43	LED	Streetlight (Warm White)	4464	100			

CCT = Correlated Color Temperature, CRI = Color Rendering Index, LE = Luminous Efficacy. CCT and CRI derived from the high resolution spectra acquired of each lamp. LE is based on manufacturer reported data. References for the Lumens and Watts are listed in a spreadsheet available at http://www.ngdc.noaa.gov/dmsp/nightsat.html.

**Table 2.** Band combinations tested-with their abbreviations.

Set 1:	Photopic (P)
Set 2:	Photopic, Scotopic (PS)
Set 3:	Photopic, Scotopic, “meltopic” (PSM)
Set 4:	TM1, Photopic, TM3 (1P3)
Set 5:	TM1, Photopic, TM3, NIR (1P3N)
Set 6:	TM1, TM2, TM3 (123)
Set 7:	TM1, TM2, TM3. NIR (123N)
Set 8:	TM1, Orange, NIR (1ON)
